# Evolutionary Perspective and Theories on the Possible Origin of SARS-CoV-2

**DOI:** 10.7759/cureus.18981

**Published:** 2021-10-22

**Authors:** Amanj A Saeed

**Affiliations:** 1 School of Medicine, University of Sulaimani, Sulaymaniyah, IRQ

**Keywords:** sars-cov-2, angiotensin-converting enzyme 2, phylogenetic analysis, vipr, zoonotic transfer

## Abstract

Objective: From the currently available next-generation sequencing data, we have tried to analyze different theories on the origin of severe acute respiratory syndrome coronavirus 2 (SARS-CoV-2) and thereby to identify the origin of its intermediate host. Genome sequence-based phylogenetic analysis and multiple sequence alignment were performed.

Methods: We used the Virus Pathogen Resource (ViPR) platform for phylogenetic analysis and the MUltiple Sequence Comparison by Log- Expectation (MUSCLE) algorithm for whole genome sequence alignment of SARS-CoV-2, severe acute respiratory syndrome coronavirus (SARS-CoV), BJ01, Middle East respiratory syndrome coronavirus (MERS-CoV), bat coronavirus RaTG13, and pangolin coronavirus.

Results: From these two analyses, we have found that RaTG13 is the closest relative to SARS-CoV-2 and not the pangolin coronavirus in spite of having sequence homology-based similarity in the genes. Comparing the RNA-dependent RNA polymerase (RdRp) and interacting spike (S) protein that interacts directly with the host human angiotensin-converting enzyme 2 (hACE2), the bat coronavirus RaTG13 was found to be the closest relative to SARS-CoV-2. Through multiple sequence alignment of the amino acid sequences, we found the furin-like cleavage site RRARS only in SARS-CoV-2 at the junction of the two subunits S1/S2 of the spike protein.

Conclusions: The possible zoonotic transfer that has happened in SARS-CoV-2 seems to not be from the pangolin, but RaTG13 remains closest relative to SARS-CoV-2. Further studies, such as systematic reviews of the literature and meta-analyses, are needed to reach a conclusion regarding the evolutionary trajectory of the SARS-CoV-2 outbreak.

## Introduction

The novel coronavirus, also known as 2019-nCoV or severe acute respiratory syndrome coronavirus 2 (SARS-CoV-2), emerged in December 2019 at Wuhan, China, and has been a pandemic causing 4.2M deaths worldwide [1]. The infection rapidly spread all over the world; on March 11th, 2020, the state of the pandemic was officially declared by the WHO [2]. Coronaviruses are a large family of enveloped viruses, with a positive-sense single-stranded RNA genome of about 30 kb. Both SARS-CoV-2 and severe acute respiratory syndrome coronavirus (SARS-CoV) belong to β-genus [3,4]. In spite of the sequence similarities between SARS-CoV-2 and SARS-CoV, correlations between their pandemic potential and biological properties are still to be deciphered [5]. SARS-CoV-2 is a recent infective human coronavirus apart from previously known SARS-CoV and Middle East respiratory syndrome coronavirus (MERS-CoV).

SARS-CoV-2 is an enveloped, non-segmented, positive-sense single-strand RNA virus whose genome codes for several structural proteins such as membrane (M) protein, spike (S) protein, nucleocapsid (N) proteins, envelope (E) protein, and 16 putative nonstructural proteins (nsps) encoded by the replicase complex (orf1ab) [6-8]. The genome also consists of several unidentified nonstructural open reading frames along with a 5′-untranslated region (UTR) and 3′-UTR [9-12].

RNA coronaviruses, including SARS-CoV-2, have a remarkably high genetic variability, which results from continuous convergent mutations that help the pathogen to adapt to the new host, and the eventual emergence of new variants with the capacity to spread more rapidly and may escape immune recognition and vaccination. The viral RNA-dependent RNA polymerase (RdRP) uses its genome to produce a negative-strand RNA intermediate, which serves as a template for the synthesis of positive-strand genomic RNA [9-12]. Recent studies have revealed the unique complexity in the coding capacity of SARS-CoV-2 genome, illustrating 23 novel viral open reading frames serving as accessory proteins or regulatory unit that controls the requisite production of different viral proteins [13]. The process of protein synthesis of coronaviruses is characterized by the generation of sub-genomic transcripts that contain a 5′ leader fused to different segments from the 3′ end of the virus genome; each unique fusion is orchestrated by a six to seven nucleotide core sequence called a transcription regulatory sequence (TRS). They are located at the 3′ end of the leader sequence that precedes each viral gene [3]. Comparing host-pathogen interaction between the human host and coronavirus, it is known that both SARS-CoV and SARS-CoV-2 use the same cell entry receptor, human angiotensin-converting enzyme 2 (hACE2). It has been analyzed that the binding affinity with the protein hACE2 and the entire SARS-CoV-2 is comparable or lower to that of the SARS-CoV spike, suggesting the possibility that the receptor-binding domain (RBD) of SARS-CoV-2 is more potent though less exposed as compared to the RBD of SARS-CoV [14].

Since the outbreak of the SARS-CoV-2 infection, several questions have been raised about the origin of the virus and the possible role of an animal as an intermediate host. However, many of the previously known cases were epidemiologically linked to the seafood “Wet” market [15]. These raised the suspicion of a zoonotic infection that had earlier infected the human population as the Middle East respiratory syndrome (MERS) in the year 2012 and severe acute respiratory syndrome (SARS) in 2002. However, convincing and reliable evidence to confirm the exact origin and the evolutionary pathway of SARS-CoV-2 is yet to be identified.

In this article, we have tried to analyze different theories on the origin of SARS-CoV-2 and its intermediate host from the currently available genome sequencing data, which will help in blocking interspecies transmission.

## Materials and methods

Phylogenetic analysis and multiple sequence alignment

All the genome sequence data of human coronavirus SARS-CoV-2, SARS-CoV, bat RaTG13, and pangolin coronavirus were downloaded from the Virus Pathogen Resource (ViPR) database (https://www.viprbrc.org/brc/home.spg?decorator=vipr). The strain name, GenBank sequence accession number, sequence length, host from which the genome sequence has been identified, and the country of the origin have been tabulated in Table 1. Spike protein amino acid sequences of SARS-CoV-2, SARS-CoV, RaTG13, pangolin coronavirus PCoV_GX, and MERS-CoV were downloaded from the National Center for Biotechnology Information (NCBI) database in FASTA format (https://www.ncbi.nlm.nih.gov/sars-cov-2/). The strain name corresponding to the NCBI protein accession number of the spike protein sequences has been tabulated in Table 2.

**Table 1:** Genome sequence of coronaviruses included in generation of the phylogenetic tree on the basis of whole genome. Genome sequences were obtained from the Virus Pathogen Resource (ViPR) database.

Strain Name	GenBank Accession	Sequence Length	GenBank Host	Country
PCoV_GX-P5L	MT040335	29806	Manis javanica (Malayan pangolin)	China
PCoV_GX-P5E	MT040336	29802	Manis javanica (Malayan pangolin)	China
PCoV_GX-P2V	MT072864	29795	Pangolin	China
PCoV_GX-P3B	MT072865	29801	Pangolin	China
PCoV_GX-P4L	MT040333	29805	Manis javanica (Malayan pangolin)	China
BJ01	AY278488	29725	Homo sapiens	China
RaTG13	MN996532	29855	Rhinolophus affinis	China
SARS-CoV-2/human/CHN/OS1/2020	MT407654	29817	Homo sapiens	China
SARS-CoV-2/human/AUS/VIC1562/2020	MT612183	29814	Homo sapiens	Australia
SARS-CoV-2/human/DEU/NRW-09/2020	MT582491	29782	Homo sapiens	Germany
SARS-CoV-2/human/IND/GBRC162a/20	MT576048	29800	Homo sapiens	India

**Table 2:** Spike protein sequence included in generation of the phylogenetic tree on the basis of surface glycoprotein synthesis. The protein sequence was obtained from the National Center for Biotechnology Information (NCBI) database as FASTA format, then used for phylogenetic tree and sequence alignment through the Virus Pathogen Resource (ViPR) platform.

Strain Name	Accession
surface glycoprotein [Severe acute respiratory syndrome coronavirus 2 ]	YP_009724390
spike glycoprotein [Bat coronavirus RaTG13]	QHR63300
spike protein [Pangolin coronavirus]	QIQ54048
spike glycoprotein [SARS coronavirus]	AAP41037
spike glycoprotein [Middle East respiratory syndrome-related coronavirus]	YP_009047204

From all the above genomic and amino acid sequences, phylogenetic analysis was performed using the ViPR tool employing the RaxML algorithm. We used model comparison tools to select the best model of evolution for the nucleotide data. We used the MUltiple Sequence Comparison by Log- Expectation (MUSCLE) multiple sequence alignment, available on ViPR, for the sequence alignment analysis from the amino acid sequences. Model compare tools were used to select the best model of evolution for the nucleotide data.

## Results

**Figure 1:**
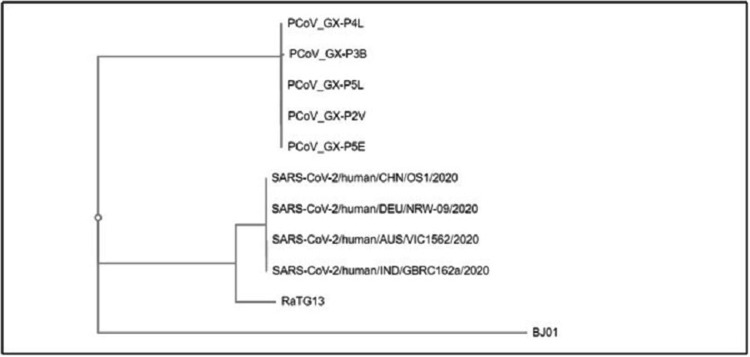
Genome sequence phylogenetic analysis. Phylogenetic relationship of severe acute respiratory syndrome coronavirus 2 (SARS-CoV-2), RaTG13 bat coronavirus, severe acute respiratory syndrome coronavirus (SARS-CoV), pangolin coronavirus, and BJ01. The whole genome sequences were obtained from the Virus Pathogen Resource (ViPR) database. The phylogenetic tree was constructed using the whole genome sequence of each strain. Phylogenetic analysis has been performed using ViPR (generate phylogenetic tree) through the RaxML algorithm. We used model compare tools to select the best model of evolution for the nucleotide data. This result indicates that SARS-CoV-2 is closely related to RaTG13 on the whole genome sequence level.

**Figure 2:**
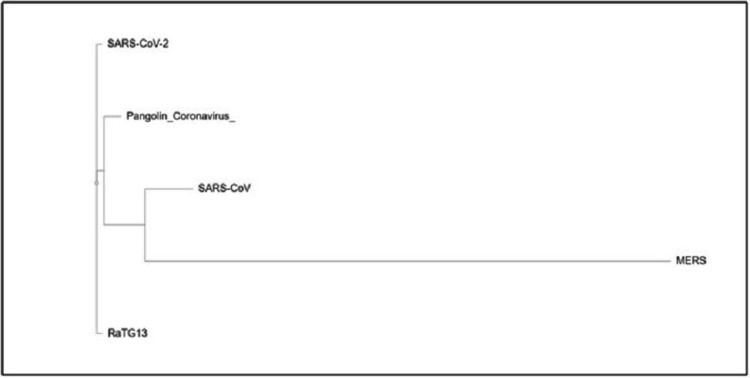
Spike protein phylogenetic analysis. Phylogenetic relationship of the spike glycoprotein amino acid sequences of severe acute respiratory syndrome coronavirus 2 (SARS-CoV-2), RaTG13 bat coronavirus, severe acute respiratory syndrome coronavirus (SARS-CoV), pangolin coronavirus and Middle East respiratory syndrome (MERS) coronavirus. The spike protein sequences were obtained from the National Center for Biotechnology Information (NCBI) database. The phylogenetic tree was constructed using the spike glycoprotein sequence obtained as FASTA format from the NCBI database. Phylogenetic analysis was performed using Virus Pathogen Resource (ViPR) through the RaxML algorithm. We used model compare tools to select the best model of evolution for the nucleotide data. This result indicates that spike protein sequences of SARS-CoV-2 and pangolin coronavirus are closely related.

**Figure 3:**
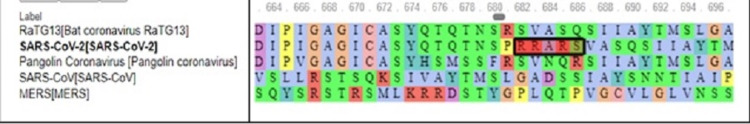
Multiple sequence alignment of the amino acid sequences. Presence of the furin cleavage site on the S1/S2 junction of severe acute respiratory syndrome coronavirus 2 (SARS-CoV-2). Protein sequences were obtained from the National Center for Biotechnology Information (NCBI) database; then multiple sequence alignment was performed on the Virus Pathogen Resource (ViPR) platform by using the MUltiple Sequence Comparison by Log-Expectation (MUSCLE) algorithm. The furin cleavage site (RRARS) is only present in the SARS-CoV-2 spike protein sequence.

Table 1 shows the features of sequences used in the whole genome-based phylogenetic analysis of human coronavirus SARS-CoV-2, SARS-CoV, bat RaTG13, and pangolin coronaviruses downloaded from the ViPR database. Table 2 shows the accession numbers of spike protein sequences included in the generation of the phylogenetic tree in the present study. Phylogenetic analysis was performed using the ViPR tool employing the RaxML algorithm and model validation was performed using compare tools. Figure 1 depicts the phylogenetic tree constructed using the whole genome sequences of viruses tabulated in Table 2. It also shows that SARS-CoV-2 strains infecting humans form a cluster together, with bat coronavirus RaTG13 as the immediate outgroup. It means SARS-CoV-2 and bat coronavirus strain RaTG13 had the most common ancestor in the recent past. Pangolin coronaviruses form a cluster together. Human coronaviruses also form a cluster together, with a common ancestor with bat coronavirus strain RaTG13. Thus, bat RaTG13 and human coronaviruses form a subclade within the pangolin clade. Figure 1 does not show the timelines despite that, it is inferred that human SARS-CoV-2 had the most common ancestor with the bat RaTG13 and not with pangolin as suggested earlier. Figure 2 shows the results of the phylogenetic analysis of spike protein of different coronavirus strains. It shows that the spike protein of the human coronavirus forms an outgroup. However, there is a similarity in spike proteins of MERS and SARS-CoV, as these are clustered together forming a sub-clade. Figure 2 shows that the S protein sequence in pangolins has more similarity to the S protein of MERSs and SARS-CoV-2 coronavirus in comparison to human coronavirus. S protein of bat coronavirus strain RaTG13 forms a distinct branch from human coronavirus, indicating that S protein evolved independently in these two groups despite high overall genome similarity. Multiple sequence alignment (MSA) using the MUSCLE tool, available at ViPR, was done for the sequence alignment analysis of amino acid sequences of the spike proteins (Table 2). It is reported that the sequence-rich human coronavirus SARS-CoV-2, SARS-CoV, bat RaTG13, and pangolin coronavirus in lysine and arginine residues create a polybasic, R-X-R/K-R motif which is attributed to virulence in pathogenic viruses. This motif distinguishes pathogenic viruses from non-pathogenic ones and is responsible for the formation of a binding and cleavage site for furin and similar enzymes. MSA was done to look out for the presence of this motif in the downloaded set of S proteins. Figure 3 depicts the result of MSA of S proteins (Table 2). SARS-CoV-2 has developed a furin cleavage site (RRARS) at S1/S2 junction that is not present in other coronaviruses (Figure 3). It is reported that this recently acquired furin-cleavage motif in the S protein of SARS-CoV-2 reduces its stability, thus facilitating a conformational change in its RBD domain for binding with the ACE2 receptor. It is speculated that higher transmissibility of SARS-CoV-2 in comparison to SARS-CoV could be a gain of function with the development of the furin-like cleavage. Figure 3 shows that this motif present in SARS-CoV-2 spike protein distinguishes it from the other coronaviruses.

## Discussion

Since the outbreak of SARS-CoV-2 infection, several questions have been raised about the origin of the virus and the possible role of the animal intermediate host; the early rise of these cases in Wuhan was associated with the seafood market raising the suspicion of a zoonotic viral infection spilled over into the human population, similar to the outbreak of other coronaviruses including SARS in 2002 and MERS in 2012 [8,10]. However, convincing and reliable evidence to confirm the exact origin and the evolutionary pathway of SARS-CoV-2 is yet to be determined. Several recent studies claimed identification of SARS-CoV-2-associated viruses in pangolins and raised the possibility of pangolin being the intermediate host of this newly emerged SARS-CoV-2 pandemic [11]. Through careful analysis and interpretation of data presented in those studies, we can see pangolins don’t seem to have any direct link to the current outbreak. The identified pangolin coronavirus genome has 85.5% - 92.4% sequence similarity with the SARS-CoV-2 genome [12], whereas RaTG13 has 96.4% sequence similarity with SARS-CoV-2 [16]. Phylogenetic analysis conducted at the whole genome level identified RaTG13, from Yunnan province of China, as the closest relative to SARS-CoV-2 (Figure 1). Several previous studies advocate that like SARS-CoV and MERS-CoV, SARS-CoV-2 probably originated in bats and ultimately spread to humans after evolving in transitional hosts [17,18]. However, SARS-CoV-2 has low similarity to the SARS-CoV and MERS-CoV genomes (Figure 1). MERS and SARS-CoV form a subclade together under a larger clade distinct from other coronaviruses (Figure 1). Despite these large genomic differences, it is noteworthy that both SARS-CoV and SARS-CoV-2 need the engagement of E-anchored S protein to enter the ACE2-expressing cells [19]. It is reported that S protein is the main contributing factor for viral tropism, transmissibility, and virulence in coronaviruses. SARS-CoV-2 has evolved very fast in the recent past by amassing mutations in the S protein, therefore scientists are predominantly focused on S protein to find the origin and evolutionary history of fast emerging coronaviruses [20-22]. It is also reported that ACE2 is recognized by the RBD of the spike of both SARS-CoV-2 and SARS-CoV [23]. One of the essential features of SARS-C0V-2 is the existence of a polybasic cleavage (RRARS) site at the junction of the S1 and S2 domain of the S protein (Figure 3), which results in a precise cleavage by furin and similar proteases and has a decisive role in establishing virulence and host range [22,24]. This motif reduces the stability, thus facilitating a conformational change in its RBD domain for binding to its receptor. It is speculated that enhanced transmissibility of SARS-CoV-2 as compared to SARS-CoV might be a gain of function as a result of the development of furin-like cleavage. Although the spike protein of SARS-CoV belongs to class 1 viral envelope glycoprotein, there is no enzymatic cleavage site to produce the S1 and S2 domains. However, when such a cleavage site was introduced to recognize furin, at putative S1-S2 junction residues of the SARS-CoV spike protein, it led to enhanced membrane fusion of the S glycoprotein. The RBD of pangolin coronavirus S protein exhibit 97.4% amino acid similarity with that of SARS-CoV-2, and both the viruses retain identical amino acids at five critical positions in RBD, however, the phylogenetic analysis of RBD at synonymous sites shows that the topological position of the pangolin coronavirus is in alignment with the rest of the viral genome, rather than proving it the closest relative of SARS-CoV-2 [11]. This analysis points to a selectively mediated and consistent convergent evolution instead of recombination. Moreover, pangolin coronaviruses identified so far lack the furin-like cleavage site at the S1/S2 junction of their spike protein which is present in SARS-CoV-2 and plays a crucial role in the emergence and rapid spread through the human population [25,26]. Despite the similarity in RBD of the S protein of SARS-CoV-2 and pangolin coronaviruses, currently available data, particularly the pattern and extent of the genome sequence differences, could not provide unequivocal evidence to support the hypothesis of pangolins being the transitional host for SARS-CoV-2 [27]. Moreover, pangolins are solitary animals with a small population size, which doesn’t seem to support precise infection dynamics to acquire polybasic cleavage sites and develop mutations in the spike protein needed for the spillover to the human host. However, the pangolin coronavirus needs to be cautiously monitored to circumvent any other possible outbreaks, as these viruses own an RBD with a high affinity to widespread mammalian species including humans [23,28]. The present study and review of the available data from pangolin and other bat coronaviruses are inadequate to navigate towards the precise evolutionary pathway of the recently emerged SARS-CoV-2; hence a long term and systemic monitoring of coronaviruses in different animal species are required, predominantly in species that share a common ecological niche with bats and pangolins. Moreover, highly professional, scientific, and fair investigation at the international level should be arranged to trace the evolutionary route of the SARS-CoV-2 outbreak.

## Conclusions

Phylogenetic analysis, followed by multiple sequence alignment of the genome sequence, is one of the best strategies in evaluating genomic lineage. The pangolin coronavirus contains high-affinity RBD towards a wide spectrum of mammalian species, including humans. However, the available data from pangolin and other bat coronaviruses could not navigate the exact evolutionary pathway of the newly emerged SARS-CoV-2. Further studies, such as systematic reviews of the literature and meta-analyses, are needed to reach a conclusion regarding the evolutionary trajectory of the SARS-CoV-2 outbreak.
